# 2,5-Dichloro-*N*-(2,3-dimethyl­phen­yl)benzene­sulfonamide

**DOI:** 10.1107/S1600536812035787

**Published:** 2012-08-23

**Authors:** Shumaila Younas Mughal, Islam Ullah Khan, William T. A. Harrison, Muneeb Hayat Khan, Muhammad Nawaz Tahir

**Affiliations:** a Materials Chemistry Laboratory, Department of Chemistry, GC University, Lahore 54000, Pakistan; b Department of Chemistry, University of Aberdeen, Meston Walk, Aberdeen AB24 3UE, Scotland; c Questioned Documents Unit, Punjab Forensic Science Agency, Home Department, Lahore, Pakistan; d Department of Physics, University of Sargodha, Punjab, Pakistan.

## Abstract

In the title compound, C_14_H_13_Cl_2_NO_2_S, the dihedral angle between the aromatic rings is 62.21 (7)° and the C—S—N—C group adopts a *gauche* conformation [torsion angle = 60.22 (17)°]. In the crystal, N—H⋯O hydrogen bonds link the mol­ecules into *C*(4) chains propagating in [010]. A short inter­molecular Cl⋯O contact of 3.1115 (17) Å is seen.

## Related literature

For related structures, see: Mughal *et al.* (2012*a* ▶,*b* ▶).
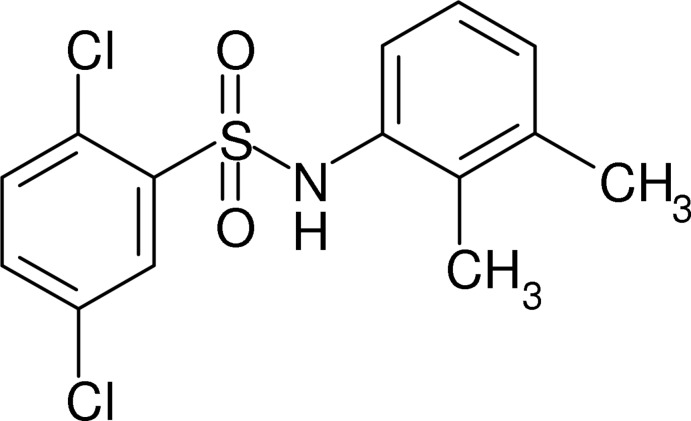


## Experimental

### 

#### Crystal data

C_14_H_13_Cl_2_NO_2_S
*M* *_r_* = 330.21
Orthorhombic, 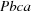

*a* = 13.0069 (11) Å
*b* = 10.0775 (9) Å
*c* = 22.408 (2) Å
*V* = 2937.2 (4) Å^3^
*Z* = 8
Mo *K*α radiation
μ = 0.58 mm^−1^
*T* = 296 K
0.30 × 0.20 × 0.18 mm

#### Data collection

Bruker APEXII CCD diffractometer
23820 measured reflections
3260 independent reflections
2377 reflections with *I* > 2σ(*I*)
*R*_int_ = 0.045

#### Refinement

*R*[*F* ^2^ > 2σ(*F* ^2^)] = 0.036
*wR*(*F* ^2^) = 0.090
*S* = 0.98
3260 reflections
186 parameters
H atoms treated by a mixture of independent and constrained refinement
Δρ_max_ = 0.26 e Å^−3^
Δρ_min_ = −0.31 e Å^−3^

### 

Data collection: *APEX2* (Bruker, 2007 ▶); cell refinement: *SAINT* (Bruker, 2007 ▶); data reduction: *SAINT*; program(s) used to solve structure: *SHELXS97* (Sheldrick, 2008 ▶); program(s) used to refine structure: *SHELXL97* (Sheldrick, 2008 ▶); molecular graphics: *ORTEP-3* (Farrugia, 1997 ▶); software used to prepare material for publication: *SHELXL97*.

## Supplementary Material

Crystal structure: contains datablock(s) global, I. DOI: 10.1107/S1600536812035787/bt6820sup1.cif

Structure factors: contains datablock(s) I. DOI: 10.1107/S1600536812035787/bt6820Isup2.hkl

Supplementary material file. DOI: 10.1107/S1600536812035787/bt6820Isup3.cml

Additional supplementary materials:  crystallographic information; 3D view; checkCIF report

## Figures and Tables

**Table 1:** Hydrogen-bond geometry (Å, °)

*D*—H⋯*A*	*D*—H	H⋯*A*	*D*⋯*A*	*D*—H⋯*A*
N1—H1⋯O1^i^	0.87 (2)	2.14 (2)	2.975 (2)	162.9 (19)

Symmetry code: (i) 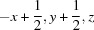.

**Fig. 1.**
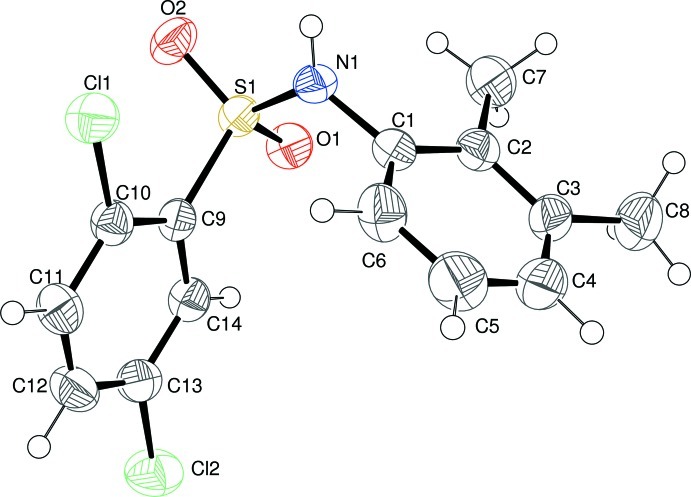
The molecular structure of (I), showing displacement ellipsoids at the 50% probability level.

**Fig. 2.**
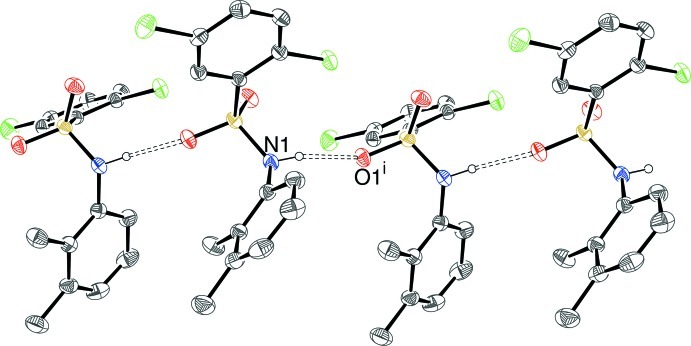
Fragment of a C(4) chain in the crystal of (I), with N—H···O hydrogen bonds shown as double-dashed lines. All C-bound H atoms omitted for clarity. Symmetry code: (i) 1/2 - x, y + 1/2, z.

## supplementary crystallographic information

### Comment

The title compound, (I), (Fig. 1) was examined as part of our ongoing interest in the structural chemistry of sulfonamides (Mughal *et al.*, 2012*a*,*b*).

The dihedral angle between the C1—C6 and C9—C14 benzene rings in (I) is 62.21 (7)°. The C1—N1—S1—C9 linkage adopts a near-ideal *gauche* conformation [torsion angle = 60.22 (17)°] and the bond-angle sum about N1 (H atom freely refined) is 349°, possibly suggesting a hybridization state intermediate between *sp*^2^ and *sp*^3^, which was also observed in a related compound (Mughal *et al.*, 2012*a*). The largest bond angle at the distorted tetrahedral S atom is O1—S1—O2 [119.69 (9)°], which again is typical for this class of compound (Mughal *et al.*, 2012*b*).

In the crystal, the molecules are linked by N—H···O hydrogen bonds (Table 1) to generate C(4) chains propagating in [010]: adjacent molecules are in the chain are generated by glide-symmetry. A similar chain occurs in *N*-(2,3-dihydro-1,4-benzodioxin-6-yl)-4-fluorobenzenesulfonamide (Mughal *et al.*, 2012*a*), although in this case, adjacent molecules are generated by translational symmetry. Conversely, in 2,5-dichloro-*N*-(3-methylphenyl)benzenesulfonamide (Mughal *et al.*, 2012*b*), pairs of N—H···O interactions lead to inversion dimers in the crystal.

Any aromatic π-π stacking in (I), if it exists at all, must be extremely weak, as the shortest inter-centroid ring separation is 4.0550 (12) Å between inversion-symmetry related C9–C14 rings (slippage = 1.906 Å). A short intermolecular Cl···O contact of 3.1115 (17) Å occurs (van der Waals' radius sum for these species = 3.27 Å).

### Experimental

0.1 g of 2,3 dimethyl aniline was dissolved in 15 ml dichloromethane and 0.2 g of 2,5-dichloro benzene sulfonylchloride was added: the mixture was stirred at room temperature overnight with the pH maintained at 8–9 with triethyamine. On completion of reaction (after TLC) the mixture was poured into a separating flask and 1 *M* HCl solution added. The lower DCM layer was separated and the solvent was allowed to evaporate at room temperature. Brown blocks of (I) were recrystallized from acetonitrile solution at room temperature in 96% yield.

### Refinement

The N-bond H atom was located in a difference map and its position was freely refined. The C-bound H atoms were placed in calculated positions (C—H = 0.93–0.97 Å) and refined as riding. The constraint *U*_iso_(H) = 1.2*U*_eq_(C,N) or 1.5*U*_eq_(methyl C) was applied. The methyl groups were allowed to rotate, but not to tip, to best fit the electron density.

### Crystal data

**Table:**

C_14_H_13_Cl_2_NO_2_S	*D*_x_ = 1.494 Mg m^−^^3^
*M**_r_* = 330.21	Mo *K*α radiation, λ = 0.71073 Å
Orthorhombic, *P**b**c**a*	Cell parameters from 1890 reflections
*a* = 13.0069 (11) Å	θ = 4.2–21.6°
*b* = 10.0775 (9) Å	µ = 0.58 mm^−^^1^
*c* = 22.408 (2) Å	*T* = 296 K
*V* = 2937.2 (4) Å^3^	Block, colourless
*Z* = 8	0.30 × 0.20 × 0.18 mm
*F*(000) = 1360	

### Data collection

**Table:**

Bruker APEXII CCD diffractometer	2377 reflections with *I* > 2σ(*I*)
Radiation source: fine-focus sealed tube	*R*_int_ = 0.045
Graphite monochromator	θ_max_ = 27.2°, θ_min_ = 1.8°
ω scans	*h* = −16→15
23820 measured reflections	*k* = −12→12
3260 independent reflections	*l* = −28→28

### Refinement

**Table:**

Refinement on *F*^2^	Primary atom site location: structure-invariant direct methods
Least-squares matrix: full	Secondary atom site location: difference Fourier map
*R*[*F*^2^ > 2σ(*F*^2^)] = 0.036	Hydrogen site location: inferred from neighbouring sites
*wR*(*F*^2^) = 0.090	H atoms treated by a mixture of independent and constrained refinement
*S* = 0.98	*w* = 1/[σ^2^(*F*_o_^2^) + (0.0396*P*)^2^ + 1.0864*P*] where *P* = (*F*_o_^2^ + 2*F*_c_^2^)/3
3260 reflections	(Δ/σ)_max_ = 0.001
186 parameters	Δρ_max_ = 0.26 e Å^−^^3^
0 restraints	Δρ_min_ = −0.31 e Å^−^^3^

### Special details

**Table:**

Geometry. All e.s.d.'s (except the e.s.d. in the dihedral angle between two l.s. planes) are estimated using the full covariance matrix. The cell e.s.d.'s are taken into account individually in the estimation of e.s.d.'s in distances, angles and torsion angles; correlations between e.s.d.'s in cell parameters are only used when they are defined by crystal symmetry. An approximate (isotropic) treatment of cell e.s.d.'s is used for estimating e.s.d.'s involving l.s. planes.
Refinement. Refinement of *F*^2^ against ALL reflections. The weighted *R*-factor *wR* and goodness of fit *S* are based on *F*^2^, conventional *R*-factors *R* are based on *F*, with *F* set to zero for negative *F*^2^. The threshold expression of *F*^2^ > σ(*F*^2^) is used only for calculating *R*-factors(gt) *etc*. and is not relevant to the choice of reflections for refinement. *R*-factors based on *F*^2^ are statistically about twice as large as those based on *F*, and *R*- factors based on ALL data will be even larger.

### Fractional atomic coordinates and isotropic or equivalent isotropic displacement parameters (Å^2^)

**Table:**

	*x*	*y*	*z*	*U*_iso_*/*U*_eq_	
C1	0.36432 (15)	0.2392 (2)	0.27761 (9)	0.0380 (5)	
C2	0.34175 (15)	0.1626 (2)	0.22779 (9)	0.0403 (5)	
C3	0.41472 (17)	0.1589 (2)	0.18170 (10)	0.0471 (6)	
C4	0.50361 (19)	0.2333 (3)	0.18622 (11)	0.0607 (7)	
H1A	0.5523	0.2287	0.1559	0.073*	
C5	0.52163 (19)	0.3142 (3)	0.23476 (12)	0.0648 (7)	
H13	0.5802	0.3670	0.2362	0.078*	
C6	0.45235 (17)	0.3159 (2)	0.28092 (11)	0.0510 (6)	
H14	0.4645	0.3685	0.3143	0.061*	
C7	0.24248 (18)	0.0871 (3)	0.22183 (11)	0.0593 (7)	
H11A	0.2014	0.1006	0.2569	0.089*	
H11B	0.2570	−0.0058	0.2174	0.089*	
H11C	0.2057	0.1183	0.1874	0.089*	
C8	0.3960 (2)	0.0772 (3)	0.12657 (11)	0.0689 (8)	
H12A	0.4552	0.0817	0.1011	0.103*	
H12B	0.3371	0.1111	0.1057	0.103*	
H12C	0.3838	−0.0134	0.1377	0.103*	
C9	0.42663 (14)	0.14693 (19)	0.41375 (8)	0.0334 (4)	
C10	0.45813 (15)	0.2588 (2)	0.44538 (9)	0.0376 (5)	
C11	0.55332 (16)	0.2609 (2)	0.47257 (10)	0.0469 (5)	
H7	0.5742	0.3358	0.4936	0.056*	
C12	0.61758 (16)	0.1526 (2)	0.46883 (10)	0.0490 (6)	
H8	0.6814	0.1538	0.4875	0.059*	
C13	0.58658 (16)	0.0429 (2)	0.43732 (9)	0.0416 (5)	
C14	0.49226 (15)	0.0390 (2)	0.40944 (9)	0.0389 (5)	
H10	0.4727	−0.0356	0.3878	0.047*	
Cl1	0.38122 (4)	0.39770 (5)	0.45093 (3)	0.04880 (16)	
Cl2	0.66673 (5)	−0.09408 (7)	0.43314 (3)	0.0630 (2)	
S1	0.30265 (4)	0.13320 (5)	0.38004 (2)	0.03558 (14)	
N1	0.29345 (12)	0.24200 (17)	0.32771 (8)	0.0376 (4)	
H1	0.2777 (16)	0.322 (2)	0.3392 (10)	0.045*	
O1	0.30173 (10)	0.00454 (14)	0.35289 (7)	0.0443 (4)	
O2	0.22658 (11)	0.16635 (16)	0.42321 (7)	0.0487 (4)	

### Atomic displacement parameters (Å^2^)

**Table:**

	*U*^11^	*U*^22^	*U*^33^	*U*^12^	*U*^13^	*U*^23^
C1	0.0366 (10)	0.0394 (12)	0.0381 (11)	0.0035 (9)	−0.0028 (8)	0.0021 (10)
C2	0.0406 (11)	0.0416 (12)	0.0386 (12)	0.0063 (10)	−0.0032 (9)	−0.0006 (10)
C3	0.0519 (13)	0.0519 (14)	0.0375 (12)	0.0134 (11)	0.0019 (10)	0.0036 (11)
C4	0.0500 (13)	0.0814 (19)	0.0506 (15)	0.0037 (13)	0.0089 (11)	0.0181 (14)
C5	0.0498 (14)	0.0821 (19)	0.0627 (17)	−0.0222 (14)	−0.0005 (12)	0.0166 (15)
C6	0.0541 (13)	0.0544 (14)	0.0446 (13)	−0.0158 (12)	−0.0040 (11)	0.0014 (12)
C7	0.0507 (13)	0.0732 (17)	0.0540 (14)	−0.0088 (13)	−0.0089 (11)	−0.0155 (14)
C8	0.0825 (19)	0.078 (2)	0.0458 (14)	0.0175 (15)	0.0057 (13)	−0.0104 (14)
C9	0.0351 (10)	0.0356 (11)	0.0296 (10)	−0.0050 (9)	0.0024 (8)	−0.0019 (9)
C10	0.0390 (10)	0.0374 (11)	0.0365 (11)	−0.0029 (9)	−0.0006 (8)	−0.0027 (9)
C11	0.0462 (12)	0.0483 (13)	0.0461 (13)	−0.0077 (11)	−0.0079 (10)	−0.0096 (11)
C12	0.0382 (11)	0.0592 (15)	0.0497 (14)	−0.0038 (11)	−0.0091 (10)	−0.0002 (12)
C13	0.0401 (11)	0.0439 (12)	0.0407 (12)	0.0038 (10)	0.0028 (9)	0.0077 (10)
C14	0.0412 (11)	0.0373 (11)	0.0384 (11)	−0.0036 (9)	0.0018 (9)	−0.0021 (9)
Cl1	0.0479 (3)	0.0399 (3)	0.0586 (4)	0.0005 (2)	−0.0035 (2)	−0.0150 (3)
Cl2	0.0558 (4)	0.0571 (4)	0.0760 (4)	0.0165 (3)	−0.0020 (3)	0.0082 (3)
S1	0.0320 (2)	0.0373 (3)	0.0374 (3)	−0.0063 (2)	0.0019 (2)	−0.0072 (2)
N1	0.0382 (9)	0.0360 (9)	0.0386 (9)	0.0041 (8)	0.0001 (7)	−0.0077 (8)
O1	0.0438 (8)	0.0374 (8)	0.0515 (9)	−0.0084 (7)	−0.0015 (7)	−0.0112 (7)
O2	0.0393 (8)	0.0595 (10)	0.0473 (9)	−0.0077 (7)	0.0115 (7)	−0.0117 (8)

### Geometric parameters (Å, º)

**Table:**

C1—C6	1.384 (3)	C8—H12C	0.9600
C1—C2	1.388 (3)	C9—C14	1.386 (3)
C1—N1	1.453 (3)	C9—C10	1.394 (3)
C2—C3	1.403 (3)	C9—S1	1.786 (2)
C2—C7	1.505 (3)	C10—C11	1.380 (3)
C3—C4	1.381 (3)	C10—Cl1	1.725 (2)
C3—C8	1.505 (3)	C11—C12	1.377 (3)
C4—C5	1.379 (4)	C11—H7	0.9300
C4—H1A	0.9300	C12—C13	1.372 (3)
C5—C6	1.372 (3)	C12—H8	0.9300
C5—H13	0.9300	C13—C14	1.377 (3)
C6—H14	0.9300	C13—Cl2	1.732 (2)
C7—H11A	0.9600	C14—H10	0.9300
C7—H11B	0.9600	S1—O2	1.4235 (14)
C7—H11C	0.9600	S1—O1	1.4323 (14)
C8—H12A	0.9600	S1—N1	1.6098 (18)
C8—H12B	0.9600	N1—H1	0.87 (2)
			
C6—C1—C2	121.9 (2)	H12B—C8—H12C	109.5
C6—C1—N1	118.21 (19)	C14—C9—C10	119.30 (18)
C2—C1—N1	119.86 (18)	C14—C9—S1	117.77 (15)
C1—C2—C3	117.6 (2)	C10—C9—S1	122.90 (15)
C1—C2—C7	122.27 (19)	C11—C10—C9	120.01 (19)
C3—C2—C7	120.1 (2)	C11—C10—Cl1	118.46 (16)
C4—C3—C2	119.9 (2)	C9—C10—Cl1	121.52 (15)
C4—C3—C8	119.5 (2)	C12—C11—C10	120.4 (2)
C2—C3—C8	120.6 (2)	C12—C11—H7	119.8
C5—C4—C3	121.4 (2)	C10—C11—H7	119.8
C5—C4—H1A	119.3	C13—C12—C11	119.42 (19)
C3—C4—H1A	119.3	C13—C12—H8	120.3
C6—C5—C4	119.4 (2)	C11—C12—H8	120.3
C6—C5—H13	120.3	C12—C13—C14	121.2 (2)
C4—C5—H13	120.3	C12—C13—Cl2	119.53 (17)
C5—C6—C1	119.7 (2)	C14—C13—Cl2	119.27 (18)
C5—C6—H14	120.1	C13—C14—C9	119.7 (2)
C1—C6—H14	120.1	C13—C14—H10	120.2
C2—C7—H11A	109.5	C9—C14—H10	120.2
C2—C7—H11B	109.5	O2—S1—O1	119.69 (9)
H11A—C7—H11B	109.5	O2—S1—N1	106.47 (9)
C2—C7—H11C	109.5	O1—S1—N1	107.85 (9)
H11A—C7—H11C	109.5	O2—S1—C9	108.78 (9)
H11B—C7—H11C	109.5	O1—S1—C9	104.91 (9)
C3—C8—H12A	109.5	N1—S1—C9	108.81 (9)
C3—C8—H12B	109.5	C1—N1—S1	120.16 (14)
H12A—C8—H12B	109.5	C1—N1—H1	113.4 (15)
C3—C8—H12C	109.5	S1—N1—H1	115.5 (15)
H12A—C8—H12C	109.5		
			
C6—C1—C2—C3	3.8 (3)	Cl1—C10—C11—C12	−179.43 (18)
N1—C1—C2—C3	−177.39 (18)	C10—C11—C12—C13	0.6 (3)
C6—C1—C2—C7	−175.2 (2)	C11—C12—C13—C14	−0.1 (3)
N1—C1—C2—C7	3.6 (3)	C11—C12—C13—Cl2	−179.36 (17)
C1—C2—C3—C4	−2.0 (3)	C12—C13—C14—C9	−0.8 (3)
C7—C2—C3—C4	177.0 (2)	Cl2—C13—C14—C9	178.45 (15)
C1—C2—C3—C8	179.5 (2)	C10—C9—C14—C13	1.2 (3)
C7—C2—C3—C8	−1.5 (3)	S1—C9—C14—C13	−176.79 (16)
C2—C3—C4—C5	−1.5 (4)	C14—C9—S1—O2	127.48 (16)
C8—C3—C4—C5	177.1 (2)	C10—C9—S1—O2	−50.46 (19)
C3—C4—C5—C6	3.3 (4)	C14—C9—S1—O1	−1.71 (18)
C4—C5—C6—C1	−1.5 (4)	C10—C9—S1—O1	−179.65 (16)
C2—C1—C6—C5	−2.0 (3)	C14—C9—S1—N1	−116.91 (16)
N1—C1—C6—C5	179.1 (2)	C10—C9—S1—N1	65.14 (18)
C14—C9—C10—C11	−0.7 (3)	C6—C1—N1—S1	−93.2 (2)
S1—C9—C10—C11	177.17 (16)	C2—C1—N1—S1	87.9 (2)
C14—C9—C10—Cl1	178.49 (15)	O2—S1—N1—C1	177.30 (15)
S1—C9—C10—Cl1	−3.6 (2)	O1—S1—N1—C1	−53.06 (17)
C9—C10—C11—C12	−0.2 (3)	C9—S1—N1—C1	60.22 (17)

### Hydrogen-bond geometry (Å, º)

**Table:**

*D*—H···*A*	*D*—H	H···*A*	*D*···*A*	*D*—H···*A*
N1—H1···O1^i^	0.87 (2)	2.14 (2)	2.975 (2)	162.9 (19)

Symmetry code: (i) −*x*+1/2, *y*+1/2, *z*.
